# Women care about local knowledge, experiences from ethnomycology

**DOI:** 10.1186/1746-4269-8-25

**Published:** 2012-07-18

**Authors:** Roberto Garibay-Orijel, Amaranta Ramírez-Terrazo, Marisa Ordaz-Velázquez

**Affiliations:** 1 Laboratorio de Sistemática, Ecología y Aprovechamiento de Hongos Ectomicorrízicos, Departamento de Botánica, Instituto de Biología, Universidad Nacional Autónoma de México, Circuito Exterior s/n, A.P. 70-233, C.P. 04510, Ciudad Universitaria, D.F, Mexico; 2 Jardín Botánico, Instituto de Biología, Universidad Nacional Autónoma de México, A.P. 70-614, C.P. 04510, Ciudad Universitaria, D.F, Mexico; 3 Facultad de Ciencias, Universidad Nacional Autónoma de México, A.P. 70-181, C.P. 04510, Ciudad Universitaria, D.F, Mexico

**Keywords:** Ethnomycology, Gendered local knowledge, Women, Mushroomers, Mushrooms, Use, and Management

## Abstract

Gender is one of the main variables that influence the distribution of local knowledge. We carried out a literature review concerning local mycological knowledge, paying special attention to data concerning women’s knowledge and comparative gender data. We found that unique features of local mycological knowledge allow people to successfully manage mushrooms. Women are involved in every stage of mushroom utilization from collection to processing and marketing. Local mycological knowledge includes the use mushrooms as food, medicine, and recreational objects as well as an aid to seasonal household economies. In many regions of the world, women are often the main mushroom collectors and possess a vast knowledge about mushroom taxonomy, biology, and ecology. Local experts play a vital role in the transmission of local mycological knowledge. Women participate in the diffusion of this knowledge as well as in its enrichment through innovation. Female mushroom collectors appreciate their mycological knowledge and pursue strategies and organization to reproduce it in their communities. Women mushroom gatherers are conscious of their knowledge, value its contribution in their subsistence systems, and proudly incorporate it in their cultural identity.

## Resumen

El género es una de las principales variables que afectan la distribución de los saberes locales. Nosotros realizamos una revisión bibliográfica sobre el conocimiento micológico tradicional, prestando especial atención a los datos sobre conocimiento local de mujeres y datos comparativos entre géneros. Encontramos que el conocimiento micológico tradicional tiene características únicas que le permiten a la gente hacer un manejo adecuado de los hongos silvestres. Las mujeres se encuentran involucradas en todas las fases del uso de los hongos; desde la recolección y el procesamiento, hasta la comercialización. El conocimiento micológico tradicional incluye el uso de hongos como comida, medicina y objetos recreativos, así como un soporte de la economía familiar. En muchas regiones del mundo, las mujeres son generalmente las principales recolectoras de hongos y poseen un vasto conocimiento sobre la taxonomía local, biología y ecología de estos organismos. Los expertos locales juegan un rol vital en la transmisión del conocimiento micológico tradicional. Las mujeres, en particular, participan tanto en la difusión de estos conocimientos, como en su enriquecimiento a través de la innovación. Las recolectoras de hongos aprecian su conocimiento y buscan estrategias y organización para reproducirlo en sus comunidades. Las hongueras están consientes de su conocimiento, valoran la contribución que éste hace a su sistema de subsistencia y lo incorporan con orgullo a su identidad cultural.

Palabras clave: Etnomicología, conocimiento local con perspectiva de género, mujeres, hongueras, hongos, uso y aprovechamiento.

## Review

Gender is one of the main variables that influence local knowledge distribution [1]. It acts at two levels. The first is a consequence of culturally assigned roles for men and women and is known as “gendered knowledge”. Obvious examples include childcare and cooking. The second is derived from division of labor as a result of biological differences between men and women. Even while both engage in major activities, gender stratification occurs often linked to specific techniques or species than to participation itself [2]. However, most ethnobiological studies do not include gender comparisons. Reviews indicate that this lack of gender consciousness results in three kinds of errors: biased research design which causes omission; imbalanced analysis resulting in erroneous interpretation; and unreliability of sources which can lead to erroneous conclusions [1,3]. However, these papers make no reference to ethnomycological knowledge. While there are hardly any gender studies in ethnomycology, many give data on gender differences (Table 1). In contrast to ethnobotanical and ethnozoological knowledge, women are typically involved in all the processes of wild edible mushroom management.

**Table 1:** Ethnomycological studies with gendered data

**Ref**	**Place**	**Results**
16	Chiapas, Mex	Men collect in forests while women collect closest to their houses.
27	Hungary	Most mushroom vendors in markets are women.
34	Ozumba, Mex	Women are the main mushroom collectors and vendors. Mothers teach children to distinguish the mushrooms in “good ones” and “bad ones”.
36	South Cameroon	Men collect in forests while women collect closest to their houses. Mothers encourage children to foray for mushrooms. Mushrooms allow women to become economically independent, acquire essentials goods, and complement their diet.
38	Southeastern Poland	Men are slightly more involved in mushroom gathering than women. Sex differences in knowledge transmission are slight.
39	Southeast Asia	In most cases the women do more gathering of mushrooms than men.
40	Eastern Indonesia	Women are the main mushroom collectors. Men and women have a comparable TMK.
41	Burkina Faso	Women are the main mushroom collectors and vendors. Women have a more profound TMK than men.
42	Guyana	Women are the main mushroom collectors engaging in premeditated mushrooming; meanwhile men are only ‘opportunistic’ collectors during hunting trips. Women have a more profound TMK than men.
43	Bahrain	Women are the main mushroom collectors and vendors. They play an almost exclusive role in developing the techniques for the consumption or storage of useful mushrooms. Mushrooms allow women to gain money, become economically independent, acquire essentials goods, and complement their diet.
44	Toluca, Mex	Women are the main mushroom collectors and vendors. Women often manage the income resulting from mushroom sale. Mothers teach children to distinguish the mushrooms in “good ones” and “bad ones”.
45	Nigeria	Women are the main mushroom collectors.
46	Upper-Shaba, Zaire	Women are the main mushroom collectors.
47	Chiapas, Mex	Women are the main mushroom collectors. Men and women have a comparable TMK. Mothers teach children to distinguish the good and the bad mushrooms.
48	Australia	Women are the main mushroom collectors.
49	Nexapa, Mex	Women are the main mushroom collectors.
50	Colombia	Women are the main mushroom collectors.
51	Eastern Europe	Women are the main mushroom collectors and vendors.
52	Geneva, Italy	Collecting is an exclusive masculine activity.
53	Chiapas, Mex	Men collect in forests while women collect those mushrooms closest to their houses.
54	Chihuahua, Mex	Men and women have a comparable TMK. Women play an almost exclusive role in developing the techniques for the consumption or storage of useful mushrooms.
55	Boyaca, Colombia	Men and women have a comparable TMK.
58	Benin	Men are more knowledgeable than women.
59	Tlaxcala, Mex	Women’s mushrooming routes are more energy-efficient, allowing them to gather the same amount and a greater variety of wild mushrooms in smaller and more accessible areas of the forest. Most mushroom vendors in markets are women.
60	Tlaxcala, Mex	Most mushroom vendors in markets are women. Mothers encourage children to foray for mushrooms.
61	Hidalgo, Mex	Men target some of the most desirable species.
62	Anatolia, Turkey	Women tend to collect in groups reinforcing social networks, in contrast to men who are solitary collectors.
63	Oaxaca, Mex	Descriptions of rituals indicate that the collectors usually are young virgin women or shamans of indistinct gender.
64	Puebla, Mex	Women play an almost exclusive role in developing and refining the techniques for the consumption or storage of useful mushrooms.
65	Mex tropics	Men guide customers to foraging for hallucinogenic species.
69	Hidalgo, Mex	Most mushroom vendors in markets are women.
70, 72	Sierra Nevada, Mex	Most mushroom vendors in markets are women. Mushrooms are more important for poor women’s subsistence than for men’s.
71	Poland	Mushrooms are more important for poor women’s subsistence than for men’s.
73	Oaxaca, Mex	Most mushroom vendors in markets are women.
74	Veracruz, Mex	Most mushroom vendors in markets are women.
75	Tlaxcala, Mex	Most mushroom vendors in markets are women.
80	Burundi	Women claim rights on portions of land where edible *Termitomyces* fungi fruit.

Ref: Reference; TMK: traditional mycological knowledge; Mex: Mexico.

In this paper we use the gender differences in ethnomycological knowledge to demonstrate that women indeed care about it and value their role in its permanence. To accomplish this, we carried out a bibliographical review concerning local mycological knowledge, paying special attention to data concerning women’s knowledge and comparative gender data. Because Mexico is the country where most ethnomycological surveys have been carried out [4], a third of the references come from that region. We complement our analysis with our own field observations. Additionally, we incorporate data derived from a participatory workshop organized by the Universidad Autonoma de Chapingo called the National Mushroom Gatherers Forum “Primer Foro Nacional de Hongueros”, held in Chapingo, Mexico in August 2010. This Forum brought together regional experts on local mycological knowledge “hongueros” (mushroom collectors), researchers, and students. During the workshop, mushroom gatherers discussed their role in their communities and the challenges they face. We extracted information from women’s commentaries and provide original quotes in Spanish (Additional file 1).

### The nature of local mycological knowledge

Local mycological knowledge has features that distinguish it from both zoological and botanical knowledge. Most of these arise from fungal biology, ecology, and metabolism.

**Figure 1:**
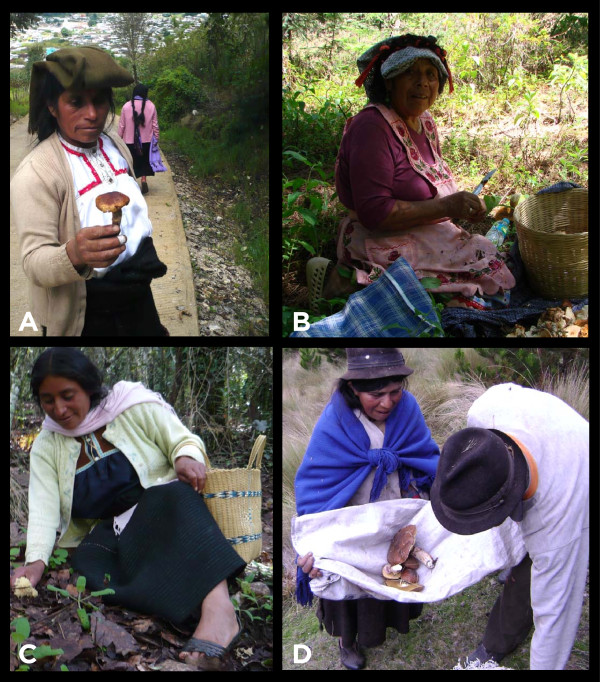
**Women collecting mushrooms and bringing them back for self-consumption or sale. 1A** - Tsotsil woman with a tricholomatoid fungi coming back to San Juan Chamula, after collecting mushrooms; Photo by Marisa Ordaz-Velázquez. **1B** - Nahua woman cleaning collected fungi; Photo by Amaranta Ramírez-Terrazo. **1C** - Tsotsil woman collecting *Ramaria* sp. from a subtropical *Quercus* spp. forest in San Juan Chamula, Chiapas, Mexico; Photo by Marisa Ordaz-Velázquez. **1D** - Kichwa descendants collecting boletes from an open high altitude pine forest in Tungurahua province, Ecuador; Photo by J. Paul Gamboa-Trujillo.

Traditional knowledge related to mushrooms is restricted to its fruit bodies, which represent the sexual stage of their life cycle [5]. There are few examples of the traditional use of their vegetative structures like mycelia or sclerotia [6]. As a consequence, only a small part of the fungal organism is subject to cultural recognition and use (Figure 1A). In contrast, plants and animals are more easily recognized as units as a whole, as well as composed of distinct structures with diverse uses. Because mushrooms have no organ development, they are often used entirely. Even while some parts of the fruit body could be removed because of bad flavor, there is rarely differential part use [7,8]. This limited structural diversity constrains the uses of mushrooms as happens with other simple organisms as non vascular plants, insects, or worms.

Mushroom production is uneven throughout the year; their appearance in temperate forests is restricted to the rain season. Consequently, the limited availability of wild mushrooms alters people behavior and fungi use during the mushroom season [9]. Given the fairly random and aggregated distribution of mushrooms, they cannot be accurately mapped from one year to the next. This differs from plant gathering, where the location of organisms is more predictable. Thus, mushroom gathering requires abilities more akin to those of hunters where greater knowledge of the habitat, niche, and morphology of useful fungi is needed in order to improve the gatherer’s success [10]. For mushroom gatherers, locating a particular species becomes more challenging. However, they usually forage in fixed “paths” or forest areas.

Foraging strategies are designed to maximize the chance of finding a group of species at a given time of the year. Species with high economic value (e.g., truffles, boletes, and chantharelles) are the exception, because even a small amount of these is worth the effort and time invested in foraging [9,11-13]. Most of these species are ectomycorrhizal, so they are not currently cultivated and their fruit bodies are scarce. Most cultivated mushrooms are saprobes while some are facultative. Because they are produced in great quantities, their prices are lower [14]. Consequently, the procurement of high-value mushrooms is highly dependent on local mycological knowledge. In contrast, technical knowledge has been developed on saprobe species.

As is the case with plants, mushrooms’ complex metabolism generates many byproducts. Among these are antibiotics, beta-glucanes, psychoactive compounds, and toxins [15] which confer them their medicinal, entheogenic, toxic, or even lethal properties. The most striking social effect is that a handful of toxic species generate awareness or even a subjacent fear when mushrooms are eaten. People will act cautiously and even refuse eating new edible mushrooms, while not so if faced with new plant or animal products. Even collectors avoid touching unrecognized species. Furthermore, these are usually grouped together in a residual category like toadstool among English speakers [12] and “jhasmuka” or “lu´” among some Mayan groups [16,17]. These terms are used to reinforce the cautious attitudes toward species not locally recognized as edible. A unique feature of local mycological knowledge, first characterized by Wasson, is the existence of mycophilic and mycophobic societies [18-20]. This is, entire cultures adopting contrasting emotions toward mushrooms ranging from an intense liking to an extreme aversion. We are not aware of any mayor botanical or animal resource producing a comparable phenomenon.

People perceive the kingdoms Animalia and Plantae almost in their entirety, whereas mushrooms are just a small fraction of the Fungi kingdom [5]. As a result, plants and animals are traditionally subdivided into life forms [21] while mushrooms are perceived as a whole [7,17]. At most, in some tropical regions they are divided in those growing on wood and those growing from the soil [22,23]. Thus, attitudes toward mushrooms are generalized, while animal or plant life forms may produce differential feelings within a society.

There are between 53,000 and 110,000 estimated mushroom species [24], of which around 2800 are used [25]. Because both ethnobotanical and ethnozoological knowledge refer to bigger cultural universes, typically nobody possesses all the information but rather there are specialists (herbalists, shamans, hunters). Mushrooms collectors around the world tend to have a wider knowledge about mushrooms not specializing in particular uses or life forms. An exception would be mazatec shamans who collect *Psilocybe* spp. for rituals [26]. In some places mushroom collectors gain social recognition. In Hungary, people who know the most about mushrooms are called “the king of mushrooms” [27], in Central Mexico are called “honguero” (mushroomer). They are usually people who became interested in wild mushrooms from a very young age and have accumulated practical and profound knowledge through the years “When I was only ten my mom taught me, and [now] my grandchildren know how to gather mushrooms as well…[I taught] two of my children and four of my grandchildren”. We have observed that several elderly women (up to 80 years old) keep collecting mushrooms until they are physically unable to (Figure 1B). During the forum, mushroom gatherers also stated “I am proud to be a mushroom gatherer, I am not ashamed to be”, “[we have to] teach our children they must not be ashamed of their parents if they are mushroom gatherers. In my house, my boy is 12 but he does like it; that is the way we are… we were born that way and we will die the same, we are mushroom gatherers”. In the United States, where mushroom collection does not have an ethnic or traditional context, the term mushroomer is applied to hobbyists who learn about mushrooms in mycological associations [28] or commercial harvesters, many of them migrants [29].

### Women care about use and management of wild mushrooms

According to the Declaration of the World Summit on Food Security, countries’ policies should focus on smallholders and rural women [30]. They are among the poorest in their societies, having limited access to land and paid labor [31]. Their contributions to food security are underestimated because household and subsistence activities are not taken into account in censuses [32]. According to FAO’s Forestry Department “Forests and trees on farms are a direct source of food and cash income for more than a billion of the world’s poorest people; they provide both staple foods and supplemental foods” [33]. Mushrooms are among the most important wild resources and, as such, they are strategic to FAO’s program on the promotion and development of non-wood forest products [25].

Wild mushroom usage involves women in every stage, from collection to processing and selling. There is no gender-specificity requirement to become a local expert. Mushroom gathering may be undertaken by either sex [34-37], while in Poland men are slightly more involved in it [38]. However in Bahrain, Mexico, Guatemala, Guyana, Nigeria, Zaire, Southeast Asia, Australia, Russia, etc., it is mainly done by women [34,39-51] (Figure 1C, 1D). To our knowledge, the only place where mushroom collecting is an exclusive masculine activity is Geneva [52]. In some tropical regions, men collect in forests while women collect those mushrooms closest to their houses [16,23,36,53]. However, in places like Guyana women could be considered the ‘champions’ of mushroom picking because they engage in active premeditated mushrooming. Meanwhile men are only ‘opportunistic’ collectors, picking up a few of the more desirable species when encountered on hunting trips [42]. It seems that in many regions of the world women are often the main collectors. This is also the case for plant gathering and managing; women have been recognized as custodians of agrobiodiversity over much of the world [3].

The distribution of local mycological knowledge is variable; those who engage actively in gathering develop a more profound knowledge on the biology, ecology, and phenology of mushrooms and are able to identify them, even at a species level [27,41]. Some studies show that men and women have a comparable knowledge [40,47,54,55]. On the other hand, certain reports indicate that women have a more profound knowledge [41,42,56] while others have found that men could be more knowledgeable than women [57,58]. Female collectors regard gathering as being an enjoyable activity: *“*I like to eat them, I like to pick them… they are very tasty”, “My son is 12 years old and he really likes to go out for mushrooms”. Even while they recognize its value as a source of additional income, they would continue to do it without necessity: “… if I won the lottery I would still take my family to the woods and we would make a day of it. Just for fun”.

Little attention has been paid to the mushroom foraging process. Route tracking studies [59] suggest that mushroom collecting is different for men and women. Women’s routes are more energy-efficient, allowing them to gather the same amount and a greater variety of wild mushrooms in smaller and more accessible areas of the forest [59,60]. Men, on the other hand, tend to target some of the most desirable species [42,61]. Women also tend to collect in groups reinforcing social networks, in contrast to men who are solitary collectors [62].

Collection of mushrooms for ritual use has specific requirements varying between ethnic groups. Descriptions of rituals indicate that the collectors usually are young virgin women or shamans of indistinct gender [63]. Mazatec healers “curanderos” using *Psilocybe* spp. for curative or divinatory purposes can be either men or women. Maria Sabina was one of the wisest healers of her time and the key informant of the first ethnomycological studies [20,26].

**Figure 2:**
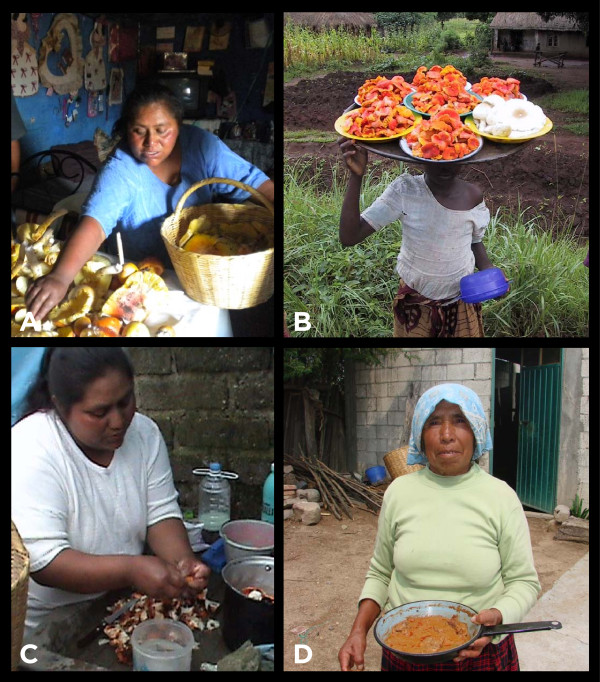
**Use and management of wild mushrooms by women. 2A** - Women selecting wild mushrooms at home; Photo by Luis Villaseñor Ibarra. **2B** - Girl carrying cantharelloid and agaricoid fungi in a bowa in her head after collecting them in the Malawi Miombo woodland; Photo by Eric Boa. **2C** - Woman preparing wild mushrooms for cooking; Photo by Luis Villaseñor Ibarra. **2D** - Woman with traditional stew “pipian de tlapitzal” made with *Gomphus floccosus*; Photo by Amaranta Ramírez-Terrazo.

Women play an almost exclusive role in developing and refining the techniques for the consumption or storage of useful mushrooms [43,54,64] (Figure 2A, 2B). This includes the culinary aspects of wild edible species, a “feminine” space throughout cultures [65] (Figure 2C, 2D). Culinary traditions are a part of cultural identity and can play a role in biodiversity conservation, since they are based on locally available biological resources. Management practices in the kitchen can also make available resources that would otherwise remain unused [3]. Mushrooms of genera including *Gyromitra* and *Gomphus,* which are slightly toxic, are boiled and rinsed two or more times before eating [66,67].

Either by sale or self-consumption, mushrooms aid the family’s economy during the rainy season. Developing methods for preserving the mushrooms allows this additional income to stretch into the dry season. Another aspect of the economical significance of processing is that it adds value to mushrooms as a marketable product [14]. Mushroom sale occurs at international, national, regional, and local levels [34]. Women can be main players in international or national markets [11,13] beyond being collectors. Regionally women may participate as middlemen selling mushrooms to companies [11,65,68]. Selling forest products, such as mushrooms is often an economic alternative for vulnerable groups, such as widows with young children, single moms, or women who are the head of their families [34,36,69]. In various places in Mexico, Africa, and Southeast Asia, many families depend on the income obtained from mushroom gathering [11,25,36,49]. Only two works [70,71] have studied the relationship between the socio-economic basis of gender inequality and mushroom use. Both concluded that in places as rural Poland and Mexico where poverty is particularly severe among women, their subsistence partly relies on mushroom sale. However, their contribution to subsistence does not necessarily relate to their status in society [3].

**Figure 3:**
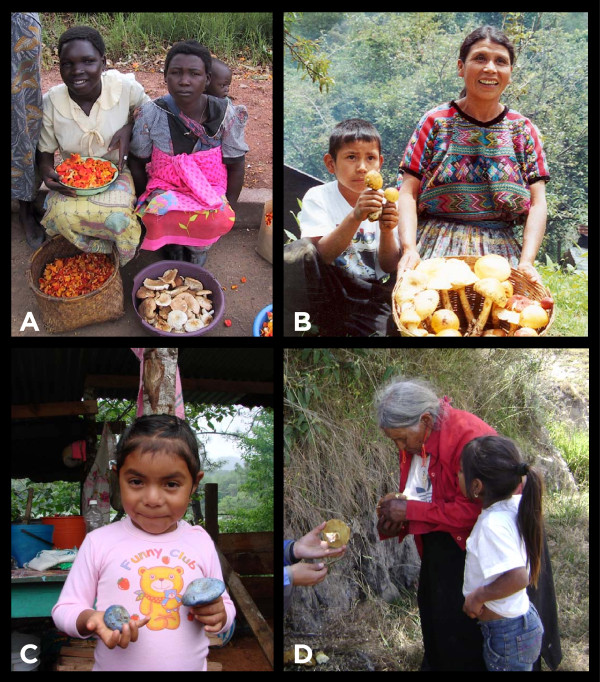
**Women and children selling mushrooms and sharing traditional mycological knowledge. 3A**- Ladies selling chanterelles and agarics on the road on Malawi; Photo by Eric Boa. **3B** - A maya Kaqchikel woman and her son selling *Amanita caesarea* complex in the town of Xetonox, Chimaltenango, Guatemala; Photo by Roberto Cáceres. **3C** - A Tojolabal descendant girl learning about edible mushrooms with some *Lactarius indigo* in her hands; Photo by Amaranta Ramírez-Terrazo. **3D** - One of the oldest informants and a little girl talking about mushrooms with the interviewer in Tungurahua, Ecuador; Photo by J. Paul Gamboa-Trujillo.

While rural gatherers usually have a modest income, middlemen in urban settings sell a considerable volume of mushrooms per season [14,34]. Locally, commercial activities concerning edible mushrooms may involve both genders. Although in temperate Mexico [34,44,59,60,64,69,72-75], Burkina Faso [41], and Hungary [27] most of the vendors in markets are women (Figure 3A, 3B). Even though men could accompany them, women establish prices and bargain with customers. In premodern Europe, particularly in east Europe, “market-women” were the main suppliers of vegetables and wild mushrooms. Nowadays, this activity continues in the Czech Republic, Ljubljana, Lithuania, Latvia, Romania, Bulgary, Poland, Ukraine, Moscow, St Petesburg, and far east, Russia [51]. In temperate regions of Mexico there is a market for hallucinogenic species used for recreational purposes. In Mexican tropical regions, where wild mushroom sale in markets is marginal [65], men are in charge of taking customers foraging for hallucinogenic species. In some places, even ceremonies for tourists are led by shamans of either sex [76].

Traditional practices are conserved in local markets where women usually barter the mushrooms that they do not sell during the day for other products. “I carried my basket filled with mushrooms and people traded tortillas, maize, water, squash, chilacayote (*Cucurbita ficifolia*) for them. Even butchers would offer us meat”. By engaging in trading, women make the most of the energy spent in collecting and optimize their income, while the remaining mushrooms are used as food [69]. Barter also has a remarkable social function as a means of constructing and strengthening social bonds [65,73]. Women often manage the income resulting from mushroom sale [44]. They care about using the revenues effectively “We save the money we make for the season when school starts… when they need something we dig into those savings”. Thus, mushrooms are a natural resource that allows women to gain some money, become economically independent, acquire essentials goods, and complement their diet [36,43].

Markets provide a space in which local knowledge is shared and transmitted [11,44]. Mushroom vendors coax potential buyers into getting the different varieties they offer by sharing recipes, telling stories, and indicating the proper procedures to ensure safe consumption. Thus, women as vendors permit urban residents to reincorporate wild mushrooms as a dietary choice.

### Women’s role in traditional knowledge dynamics

Local knowledge is intrinsically dynamic. It is constructed from a base of inherited knowledge modified by processes of enrichment (innovation, experimentation), loss (transculturation, acculturation), and transformation (syncretism) [77,78].

Rural women are particularly vulnerable in the face of global phenomena such as national policies, economical and ecological crises, food shortage, migration, urbanization, marginalization, transculturation, acculturation, environmental transformation, deforestation, and pollution [79]. Local knowledge serves them as an instrument to deal with these situations. Because local practices are developed through a continuous interaction with the environment, they also tend to be the least destructive means of appropriation. National and international conservation programs currently integrate local knowledge as a strategy to both preserve the environment and promote local identities.

When women are the primary collectors or sellers of mushrooms, they also become the main teachers of local mycological knowledge. In Poland, sex differences in knowledge transmission are slight, although fathers are most mentioned as the first teachers, and boys learn at a younger age than girls [38]. During the first years in which children learn about mushrooms, mothers often encourage them to foray for them [36,60]. When the children bring the mushrooms home they help by sorting out the “good ones” from the “bad ones” [34,44,47] (Figure 3C). In women’s words: “When I started with this mushroom thing I learned from my mom. She would tell me<<let us go searching for mushrooms>>. That is how we grew up”, “We have to teach the children from a young age; I take one of my sons, but one of the older girls does not like it. The little one does, and she is learning about mushrooms, later she will like them too”. They can also teach people from their own generation “One of my husband’s sisters did not gather or look for mushrooms. After she got together with my brother-in-law, I would tell her to join me when I went looking for mushrooms but she would say<<I do not know them>>, so I said to her<<Come on! I will teach you>>and so, she started to learn”.

However women are not always the instructors; when they marry into a family in which there is mycological knowledge, they learn from their mothers-in-law, husbands, and even their children “And now my sons […] are all married… their wives do not want to, but then they go get mushrooms themselves. They bring their children along and teach them the names of the mushrooms they find. Then the wives understand and cook them… their kids explain how. Their wives know now how to prepare mushrooms”.

Women are open to other sources of knowledge exchange such as workshops, forums, mushrooms fairs, technical seminars, and training courses. Attendants to the Mushroom Gatherers Forum said so “I took interest in the event because… we were going to talk about mushrooms, share our doubts and problems when we collect mushrooms”; “We decided to come and listen to what other collectors have to say because there are mushrooms we name one way and they name them differently. I was interested in knowing different mushrooms, listening to comments from all the collectors who are different, some coincide with us and some do not”.

Women not only transmit their inherited knowledge, they also generate new knowledge by experimentation and appropriation. In Mexico, there is evidence that women have experimented with the consumption of species not previously recognized as edible in their communities. This is accomplished through an intense observation of the biology and ecology of the suspected edible species. When these species share characteristics with other edible species, they are gathered, cooked, and served to a dog several times. If the dog survives, they taste it themselves in small quantities. When they are sure the mushroom is harmless, they serve it to their families. Also, we have observed in Tlaxcala, Mexico, the novel use of *Lyophyllum* sp. as a cosmetic product through another experimentation process. While soaking this edible mushroom, women discovered it softened their skin.

Dugan [51] has documented one of the most interesting chapters in mycology history. He states that Carolus Clusius and Franciscus van Sterbeeck, two pioneers of mycology in the sixteen and seventeen centuries, commonly obtained information about mushrooms from the wise women known as “herb-wives”. This knowledge transfer happened in a time when many women were prosecuted as witches and killed because their “illicit” botanical knowledge. Strikingly, women’s knowledge of fungi did not acquire the status of “science” until collected, systematized, and transmitted by men [51].

### Challenges women face during the mushroom appropriation process

Although mushrooms are considered a free access resource in temperate regions [22], collectors are currently being limited in their access to the forests or are required to pay a fee. “In the state of Puebla, lately they will not let us [gather]… they say we need a permit and that there is a fee. Each time we go we will have to pay… but sometimes we do not sell much. They have not told us how much it will be”. In some places of Burundi, women claim rights on portions of land where edible *Termitomyces* fungi fruit [80]. Women’s access to resources and land tenure should be explicitly recognized given their main role in agricultural and forestry practices [3]. Indiscriminate logging has destroyed forests making it more expensive to obtain mushrooms. “We pay the bus fee to get there because it is too far. It would take three or four hours if we walked. If the bus takes us there we can browse the woods all day and we get back home in the afternoon”.

In several regions a decrease in wild mushroom production has been documented. In Europe acid rain and forest soil nitrification have modified mushroom communities leading to local extinctions of several species like *Cantharellus cibarius *[81]. In Japan nematode plagues have destroyed populations of *Tricholoma matsutake,* one of the most valued edible mushrooms [82]. Local mushroom decrease is a problem collectors face “It has been about six years that you burn but there is no yield… there are no mushrooms there anymore. Very little [grows], there is not as much as before. Our grandparents used to tell how the mushrooms grew close to the houses and they found so much they did not gather it all”. They attribute this to logging, damage by livestock, change in rain patterns, and competition. “In our State [Puebla] mushrooms do not grow anymore because of logging… it does not grow nearby anymore. We have to go all the way up to *Paso de Cortés* [at least 15 km from town]”, “since cows graze there, the grass is smaller and, well, it does not [grow]. They stamp on it”.

There are dangers that are inherent to gathering activities in the forest. While gatherers recognize these, they are not an impediment to them. “In our community, many people have livestock… they do not let us look for mushrooms anymore… there are many [animals] like bulls that can gore us. They tell us they are afraid something might happen… some one has already died and they do not want that to happen again so they will not let us gather mushrooms”, “There are many snakes and scorpions, since its weedy we sometimes can not see. You are in the mountain and if the bus does not go by… venom runs fast”. Mushroom collectors, as independent workers, face these dangers without social health benefits.

They have few solutions for these trials. Nonetheless, they persist in mushroom gathering because they value this activity as a source of income and as part of their cultural identity (Figure 3D). The most important tools they have to face these challenges are their local knowledge and their skills. These allow them to adapt to changing environmental and social conditions.

## Conclusions

The unique nature of local mycological knowledge distinguishes it from both ethnobotanical and ethnozoological knowledge. This knowledge is based upon fungal biology, ecology, and metabolism, and impacts both management and perception. In rural areas, women are usually unemployed, dedicating themselves to household and subsistence activities. Mushrooms provide a source of income and nourishment, contributing to the food security of this vulnerable group. The literature indicates that, in many regions of the world, they are the principal mushroom collectors. They also play a central role on mushroom processing both for self-consumption and sale. Women are owners of a vast knowledge on mushroom taxonomy, biology, and ecology. They combine this information with their own cultural background as well as external knowledge to improve their subsistence. By doing so, they not only possess local mycological knowledge but also actively influence its evolution and transmission. Mushroom harvesting is a challenging activity that requires a deep local environmental knowledge to achieve success. Women collectors are usually not organized because they are seldom landowners and their activity is seasonal. Thus, they are unable to face challenges such as the loss of forest cover or socio-political restrictions. Women mushroom collectors are conscious of their knowledge, value its contribution in their subsistence systems and economy, and are proud of it as part of their cultural identity.

Ethnomycological studies have not been designed specifically to compare gendered knowledge and use of mushrooms. Given that women are a main force in food production and alimentary security, we strongly recommend that future studies take gender perspective into account. Design should include a stratified sampling, allowing gender comparisons. This would reveal gendered knowledge in mushroom management and permit better interpretations.

## Competing interests

Authors declare no competing interests.

## Authors’ contributions

RG-O designed the study, coordinated the analysis and interpretation of data, and wrote the manuscript. AR-T and MO-V participated in the design of the study, and made substantial contributions to the interpretation of data and manuscript writing. MO-V also reviewed the English version. All authors read and approved the final manuscript.

## Authors’ information

RG-O is an associate researcher at Universidad Nacional Autonoma de Mexico and has done work on the traditional mycological knowledge and mushroom use and management for the last ten years.

AR-T is a graduate student at Universidad Nacional Autonoma de Mexico. She is conducting a research on the cultural significance of non-edible fungi comparing two communities. She is interested in studying the role of fungi in the communities’ traditional knowledge.

MO-V is an undergraduate Biology student at Universidad Nacional Autonoma de Mexico working on local traditional knowledge of tsotsil Maya of the Highlands of Chiapas.

RG-O, MO-V, and AR-T are members of Grupo Interdisciplinario para el Desarrollo de la Etnomicologia en Mexico (GIDEM).

## Supplementary Material

Additional file 1: **Original quotes in Spanish from participants at the National Mushroomer Forum, Mexico 2010.** Quotes appear in the order in which they are included in this review. Click here for file
