# Supplementary material for: Women care about local knowledge, experiences from ethnomycology
Source: J Ethnobiol Ethnomed. 2012 Jul 18;8:25. doi: 10.1186/1746-4269-8-25 (PMC3487846; doi:10.1186/1746-4269-8-25)
Supplement: Additional file 1: — Original quotes in Spanish from participants at the National Mushroomer Forum, Mexico 2010. Quotes appear in the order in which they are included in this review. [file 1746-4269-8-25-S1.pdf]

Additional file 1. Original quotes in Spanish from participants at the National Mushroomer Forum, Mexico 2010. Quotes appear in the order in which they are found in this review.

**Quote**	**Participant**
“Ahí de 10 años me enseñó mi mamá y mis nietos también ya saben buscar hongos… [les enseñé a] dos hijos y cuatro nietos; a Domingo [uno de los hijos] lo llevaba de nueve o diez años”	Mrs. Belinda Arce; Nahua from Tlaxcala
“Yo estoy orgullosa de ser honguera, no me da pena”, “[Hay que] Enseñarle a los hijos que no se deben avergonzar de que los padres sean hongueros. En mi casa, mi niño tiene 12 años pero sí le gusta; así somos… sus abuelos, sus tíos… Así nacimos y así moriremos porque somos hongueros”	Mrs. Angeles; Nahua from Estado de México, Mexico..
“Me gusta comerlos, me gusta recogerlos... son muy sabrosos”, “Mi hijo tiene 12 y a él sí le gusta mucho ir por hongos”.	Mrs. Angeles from Estado de México, Mexico..
“…si me saco la lotería igual saco a mi familia al campo y hacemos día de campo. Ya nada más por gusto”	Mr. Anastasio from Guadalajara, Mexico
“… llevaba yo mi canasta con hongos; nos cambiaban por tortilla, nos cambiaban por maíz, agua, calabaza, chilacayota o carne, los carniceros nos ofrecían	Mrs. Rosa; Otomi from Estado de México, Mexico.
“El dinerito que vamos ganando lo guardamos para ya la temporada que van ellos a la escuela… algo que necesiten de ahí les vamos dando”	Mrs. Ángeles, Nahua from Estado de México, Mexico..
“Cuando yo empecé esto de los hongos lo aprendí de mi mamá. Me decía <<vamos a buscar los hongos>>. Así nos crecieron a nosotros”	Mrs. Rosa, Otomí from Estado de México, México.
“Así enseñándole a los niños desde que son chiquitos; yo a uno ya lo llevo pero a la otra que es más grande no le gusta, pero a otra chiquita sí le gusta ir y va conociendo y luego ella también le va a gustar.”	Mrs. Beatriz. Nahua from Estado de México, Mexico..
Tengo una concuña que no los juntaba y no los buscaban. Cuando se juntó con mi cuñado yo iba a buscar el hongo y le dije <<vamos>> y dice <<es que yo no los conozco>> y le dije <<¡Vamos! Yo te enseño>> Y los empezó a conocer.”	Mrs. Beatriz. Nahua from Estado de México, Mexico.

"Y ahora mis hijos […] están casados… sus mujeres no quieren pero ellos sí van a traer sus hongos. Jalan a sus niños y les platican cómo se llaman, los van jalando a los niños. Ya la señora también le entiende y sí lo hace… le explica el hijo. Ya sus esposas lo saben como hacer el hongo."	Mrs. Rosa. Otomi from Estado de Mexico.
"I took interest in the event because… we were going to talk about mushrooms, share our inquiries and problems when we collect mushrooms"<br><br>"We decided to come and listen to what other mushroomers have to say because there are mushrooms we name one way and they name them differently. I was interested in knowing different mushrooms, listening to comments from all the mushroomers who are different, some coincide with us and some don't".	Mrs. Beatriz. Nahua from Estado de México, Mexico.
"Estado de Puebla, son esos señores que no dejan [buscar]… que se tiene que sacar un permiso y que se tiene que pagar. Cada vez que vaya uno tiene que pagar… pero a veces no se vende bien. No nos han dicho cuanto."	Mrs. Angeles Nahua from Estado de México, Mexico..
"…pagamos nuestro pasaje para que nos lleve porque está m alejado… 3, 4 horas caminando. Si en camión nos vamos ya buscamos todo el día, nos regresamos ya en la tarde…"	Mrs. Beatriz. Nahua from Estado de México, Mexico..
"Ya hay unos seis años que quemas y ya no… por esa parte ya no hay- [Sale] poco porque ya no hay como antes muchísimo. Nuestros abuelitos nos platicaban que antes salían cerquita de la casa y encontraban mucho y no lo juntaban…"	Mrs Rosa. Otomi from Estado de Mexico.
"En nuestro estado [Tlaxcala] ya no se da el hongo por lo mismo de la tala… por ahí ya no se da. Hay que subir hasta Paso de Cortés…"	Mrs. Angeles Nahua from Estado de México, Mexico...
"como las vacas están ahí es pequeño el zacatón y pues… ya no. Ya lo machuca…"	Mrs. Rosa. Otomi from Estado de Mexico.
"Nosotros allí en nuestra comunidad, muchos que tienen su ganado ya no nos dejan buscar el hongo…hay muchos [animales]	Mrs. Beatriz. Nahua from Estado de

ahora sí que el toro que topa. [dicen] Que no nos vaya a pasar algo… un señor se murió y ya no quieren que vuelva a suceder, ya no nos dejan colectar hongos”	México, Mexico.
“Hay muchas víboras, escorpiones, que como está muy hierboso no se llega uno a fijar el pie. Está uno en el monte y el camión no pasa… el veneno corre rápido. Corremos peligro de las vacas también… al señor le enterró el cuerno y lo mató.”	Mrs. Beatriz. Nahua from Estado de México, Mexico.
Su nuera se desbarrancó, “se resbaló”.	Mrs. Belinda Arce; Nahua from Tlaxcala
